# Robert Arthur

**Published:** 1880-07

**Authors:** 


### OBITUARY.

*Robert Arthur, M. D., D. D. S.*, one of the most widely known dental practitioners in this country, died in Baltimore City, June, 1880. Dr. Arthur was the oldest graduate of the Baltimore College of Dental Surgery, having been a member of the first class, that of 1840, which assembled at the organization of this the pioneer of Dental Schools. After graduating Dr. Arthur practiced his profession in Virginia and the District of Columbia, afterwards removing to Philadelphia, where he was elected to a Professorship in the Pennsylvania College of Dental Surgery, which he held for a number of years. About the year 1857 Dr Arthur returned to Baltimore, where he has been in continuous practice up to the time of his death, which occurred from an affection of the arteries of the legs. Dr. Arthur first became noted for his manner of working adhesive gold foils, and afterwards for his views and methods concerning the prevention and treatment of dental caries, several treatises on which he published, as well as works on other dental subjects. He was also noted for his inventions, such as the disk for separating teeth, etc. We have been promised an extended sketch of Dr. Arthur's life, which will appear in a future number of this Journal.

